# Development of a sensitive competitive enzyme-linked immunosorbent assay for serodiagnosis of *Burkholderia mallei*, a Tier 1 select agent

**DOI:** 10.1371/journal.pntd.0010007

**Published:** 2021-12-21

**Authors:** Ulrich Wernery, Elaine Chan, Rekha Raghavan, Jade L. L. Teng, Ginu Syriac, Sing-Yung Siu, Marina Joseph, Man-Lung Yeung, Lilong Jia, Jian-Piao Cai, Tsz-Ho Chiu, Susanna K. P. Lau, Patrick C. Y. Woo

**Affiliations:** 1 Central Veterinary Research Laboratory, Dubai, United Arab Emirates; 2 Department of Microbiology, Li Ka Shing Faculty of Medicine, The University of Hong Kong, Hong Kong; 3 State Key Laboratory of Emerging Infectious Diseases, The University of Hong Kong, Hong Kong; 4 Carol Yu Centre for Infection, The University of Hong Kong, Hong Kong; Colorado State University, UNITED STATES

## Abstract

Glanders is a highly contagious and potentially serious disease caused by *Burkholderia mallei*, a Tier 1 select agent. In this study, we raised a monoclonal antibody (mAb) against the lipopolysaccharide (LPS) of *B*. *mallei* and developed a competitive enzyme-linked immunosorbent assay (cELISA) for *B*. *mallei* infection. Using the titrated optimal conditions of *B*. *mallei-*LPS (2 ng) for microtiter plate coating, sample serum dilution at 1:20 and 3.5 ng/μL anti-LPS mAb B5, the cutoff value of the cELISA was determined using serum samples from 136 glanders-free seronegative horses in Hong Kong. All calculated percentage inhibition (PI) values from these seronegative samples were below 39.6% inhibition (1.5 standard deviations above mean PI) and was used as the cutoff value. The diagnostic sensitivity of the developed LPS-based cELISA was first evaluated using sera from donkeys and mice inoculated with *B*. *mallei*. An increasing trend of PI values above the defined cELISA cutoff observed in the donkey and mouse sera suggested positive detection of anti-LPS antibodies. The sensitivity and specificity of the LPS-based cELISA was further evaluated using 31 serologically positive horse sera from glanders outbreaks in Bahrain and Kuwait, of which 30 were tested positive by the cELISA; and 21 seronegative horse sera and 20 seronegative donkey sera from Dubai, of which all were tested negative by the cELISA. A cELISA with high sensitivity (97.2%) and specificity (100%) for the detection of *B*. *mallei* antibodies in different animals was developed.

## Author summary

Glanders is a highly contagious and life-threatening disease caused by *Burkholderia mallei*, a Tier 1 select agent, with no available vaccine. The disease is endemic in the Middle East, Asia, Africa and South America with sporadic outbreaks and mainly occurs in horses, donkeys and mules, although it has also been reported in camels, tigers, lions, and even humans. As the bacterium is not easily isolated from clinical specimens and correct identification based on clinical signs is difficult, it is thus important to develop serological tests which can quickly diagnose *B*. *mallei* infection. In this study, we generated a monoclonal antibody against *B*. *mallei* lipopolysaccharide and used it to develop a competitive enzyme-linked immunosorbent assay (cELISA) for the serodiagnosis of *B*. *mallei* infection. The developed cELISA was optimized and evaluated using glanders-free and glanders-positive horses, donkeys and mice from Hong Kong and the Middle East, and was shown to be highly sensitive and specific for the detection of glanders in different animals. A simple and inexpensive test to allow for the early detection and diagnosis of suspected clinical cases as well as the screening of apparently asymptomatic animals will be helpful in controlling the spread and elimination of the disease.

## Introduction

Glanders is a highly contagious and potentially serious disease caused by *Burkholderia mallei*–a highly pathogenic, Gram-negative β-proteobacterium, endemic in the Middle East, Asia, Africa and South America [1–2]. Although glanders mainly occurs in horses, donkeys and mules, occasionally the bacterium also infects other mammals, such as carnivores, through ingestion of meat from sick animals. A few years ago, we reported the first case of glanders in a dromedary camel, which contracted the infection in an outbreak of glanders from horses in Bahrain [3]. In animals, glanders is usually acquired through close contact, inhalation or the ingestion of contaminated feed or water. *B*. *mallei* occasionally infects human through contact with infected animals [4–5]. Laboratory-acquired infections have also been reported [6]. Due to the high fatality rate of the disease, aerosol transmissibility of the infectious agent and small number of bacteria required to establish an infection, *B*. *mallei* has been classified as a Tier 1 select agent by the Centers for Disease Control and Prevention, USA [7].

Laboratory diagnosis of glanders can be difficult. The bacterium is often not readily isolated from clinical specimens due to its slow growth rate on standard culture media [8] and may not be correctly identified based on its clinical features even when isolated. Efforts have been spent on the development of serological tests for glanders. Since *B*. *mallei* can infect a variety of animals as well as human, the optimal serological test would be one which is able to detect *B*. *mallei* antibody from different kinds of mammals. In this study, we raised a monoclonal antibody (mAb) against the lipopolysaccharide (LPS) of *B*. *mallei* and developed a competitive enzyme-linked immunosorbent assay (cELISA) for *B*. *mallei* infection. Serologically negative serum samples from glanders-free horses in Hong Kong were used to develop and determine the cutoff value of the cELISA. The assay was then evaluated using serum samples from donkeys and mice inoculated with *B*. *mallei* and those from horses in previous glanders outbreaks in Bahrain and Kuwait.

## Materials and methods

### Ethics statement

The use of mice in this study for monoclonal antibody production was approved by the Committee on the Use of Live Animals in Teaching and Research (CULATR), The University of Hong Kong (approval number 4124–16). All the experimental procedures were performed in accordance with the International Guiding Principles for Biomedical Research Involving Animals regarding the care and use of animals.

### Serum samples

A total of 177 seronegative and 31 seropositive sera were obtained to develop and evaluate the cELISA test. The 177 seronegative sera were obtained from glanders-free horses or donkeys, including 136 horse sera from the Agricultural, Fisheries and Conservation Department (AFCD), Hong Kong and 21 horse sera and 20 donkey sera from the Central Veterinary Research Laboratory (CVRL), Dubai, The United Arab Emirates (UAE). The 31 seropositive sera were obtained from two separate glanders outbreaks in the Middle East. These included 25 horse sera from a glanders outbreak in Bahrain in year 2010–2011 [9–10] and 6 horse sera from a glanders outbreak in Kuwait in year 2019 [11]. All serum samples were confirmed as seronegative or seropositive for glanders using the complement fixation test (CFT), which served as the gold standard in this study [12].

### Animal inoculation experiments using *B*. *mallei*

Donkeys and mice were inoculated with live and non-viable preparations, respectively, of *B*. *mallei* strain MB1731, which was isolated in 2004 from the choana of a glanderous horse from Syria that was held in quarantine in UAE. The verification of MB1731 as *B*. *mallei* was confirmed using a flagellin P (*fliP*)-based PCR [13]. A standardized *B*. *mallei* inoculum was prepared by culturing MB1731 on defibrinated sheep blood agar (Oxoid Ltd, Hampshire, United Kingdom) and incubating at 37°C for 72 hrs. All colonies of MB1731 were then suspended in 0.85% NaCl. The inoculum suspension was adjusted according to the required dilution and used directly for the donkey challenge experiments. For the inoculation of mice, the adjusted inoculum suspension was further heat-inactivated at 80°C for 10 min and mixed with an equal volume of Freund’s incomplete adjuvant (Sigma).

Seven donkeys were challenged with *B*. *mallei* strain MB1731 via five routes of infection at infection doses as described in [14] and as below. Three donkeys were orally infected by direct injection to the oropharynx (two with 1 mL inoculum at concentration of 1.0 × 10^9^ CFU/mL, donkeys 1 and 2; and one with 1 mL inoculum at concentration of 1.0 × 10^8^ CFU/mL, donkey 3), one was infected by direct feeding through water (1 mL inoculum at concentration of 2.0 × 10^8^ CFU/mL, donkey 4), one was infected by direct feeding through feed (1 mL inoculum at concentration of 4.0 × 10^8^ CFU/mL, donkey 5), one was infected by nasal spray (1 mL inoculum at concentration of 1.0 × 10^8^ CFU/mL, donkey 6), and one was infected by subcutaneous injection on the left side of the neck (1 mL inoculum at concentration of 2.0 × 10^2^ CFU/mL, donkey 7). Blood samples were taken from the challenged donkeys from 6 days post infection (dpi) up to 25 dpi or until euthanized

Two female BALB/c mice were inoculated twice, at 21 days apart, with non-viable cell preparations of *B*. *mallei* MB1731 via intraperitoneal injection of 0.2 mL inoculum at concentration of 1.0 × 10^8^ CFU/mL. Blood samples were taken from the mice at 10, 25, and 37 days after the first inoculation.

### Purification of LPS

All experiments involving live *B*. *mallei* followed the approved standard operating procedures of the HKU Biosafety Level-3 facility. LPS was extracted and purified from *B*. *mallei* strain MB1731 using the LPS Extraction Kit (iNtRON Biotechnology, Korea) as per the manufacturer’s instructions with minor modifications. Briefly, bacterial cells from culture on horse blood agar grown for 3 days at 37°C were harvested and lysed in 20 mL lysis buffer. Cell clumps were dissolved by vigorous vortexing and 4 mL chloroform was added. The sample was then vortexed briefly, incubated at room temperature for 5 min and centrifuged at 11,000 × *g* for 10 min at 4°C. The top aqueous layer was transferred to a clean tube and two volumes of purification buffer were added. The sample was incubated for 10 min at -20°C and centrifuged at 11,000 × *g* for 15 min at 4°C. The LPS pellet was then washed with 70% EtOH and dissolved in 10 mM Tris-HCl buffer (pH 8.0) by vortexing and boiling for 2 min. The extracted LPS was further treated with 2.5 μg proteinase K per 1 μg LPS for 30 min at 50°C to remove contaminating proteins.

### Immunization of mice for production of mAbs

BALB/c mice were immunized five times at two-week intervals with 50 μg of purified LPS via intraperitoneal injections without adjuvant. Antibody titer to LPS was assessed using ELISA at weeks 4 and 6 post-immunization. A final boost of 50 μg LPS was injected intravenously 3 days prior to the harvesting of spleen cells. Hybridoma cell lines were then produced as previously described [15] and assessed for anti-LPS mAbs using an indirect ELISA coated with purified LPS (described below).

### Production and purification of mAbs by ascites induction

Hybridoma line (B5) positive for anti-LPS mAbs were propagated and 5 × 10^6^ cells were administered into BALB/c mice via intraperitoneal infection following priming with Freund’s incomplete adjuvant (Sigma) as previously described [15]. Ascitic fluid was collected and stored at -80°C with 0.02% sodium azide. The mAb was then purified using Protein G-Agarose (Roche) as per the manufacturer’s instructions. The subclass and light chains of the purified antibody was then determined using the Pierce Rapid Antibody Isotyping Kit Plus Kappa and Lambda for mouse (ThermoFisher) as per the manufacturer’s instructions.

### Western blot analysis

Bacterial strains were suspended in PBS, then 8 × 10^6^ cells were mixed with SDS-PAGE sample buffer and boiled for 10 min. The samples were then run by electrophoresis on a 10% SDS gel followed by semi-dry transfer onto PVDF membrane. The membranes were blocked with 5% skim milk in tris-buffered saline/Tween (TBST; 50 mM Tris, 150 mM NaCl, 0.1% Tween 20; pH 7.6) for 3 h and incubated with 1 μg/mL of B5 mAb at 4°C overnight. Goat anti-mouse IgG (H+L) cross-absorbed secondary antibody, DyLight 680 (Invitrogen) was used at 1:5,000 dilution to facilitate detection via fluorescent signal. The final development of the signal was detected using the Odyssey CLx Near-Infrared Fluorescence Imaging System (LICOR) and analyzed using the Image Studio software (LICOR).

### Specificity of mAb B5 and optimization using indirect ELISA

The specificity of mAb B5 to LPS and the optimal concentration to use for the assay was assessed using indirect ELISA. Microtiter plates (Thermo Scientific) were coated with 0 to 4 ng of purified LPS or bovine serum albumin (BSA) in 0.05 M carbonate buffer (15 mM Na_2_CO_3_, 3.5 mM NaHCO_3_) and dried at 42°C for 3 h. Wells were then blocked with blocking buffer (0.121% Tris-base, 0.2% gelatin, 2% sucrose, 0.02% thimerosal, 0.25% casein, 0.5% Tween20) at 4°C overnight. After blocking, the plates were used immediately or completely dried and stored at 4°C for up to 3 months. To assess the optimal concentration of mAb B5 to use, plates were incubated with 1.75, 3.5, and 7 ng/μL mAb B5 diluted in sample dilution buffer (1× PBS, 1% BSA, 0.1% Tween 20) at 37°C for 1 h. After incubation, the plates were washed with phosphate-buffered saline/Tween (PBST; 1× PBS, 0.5% Tween 20) and incubated with 1:1,000 anti-mouse IgG horseradish peroxidase (HRP) conjugate (ThermoFisher Scientific) diluted in enzyme dilution buffer (1× PBS, 20% fetal bovine serum, 0.5% Tween 20) at 37°C for 1 h. The plates were washed again with PBST and developed by adding 100 μL of tetramethylbenzidine (TMB) substrate (ThermoFisher Scientific) into each well. After 10 min, 0.3 M H_2_SO_4_ was used to stop the reaction and the optical density at 450 nm (OD_450_) was read using a PerkinElmer VICTOR X3 multilabel reader.

### Competitive ELISA

cELISA was performed to detect the presence of anti-LPS antibodies in serum samples. Plates were coated with 2 ng of purified LPS and blocked as per the indirect ELISA. The plates were first incubated with 50 μL of 1:20 serum sample (or unless otherwise specified) diluted in sample dilution buffer. Fifty microliters of 3.5 ng/μL mAb B5 diluted in sample dilution buffer was then added and the plates were incubated at 37°C for 1 h. After incubation, the plates were washed with PBST and incubated with 1:1,000 anti-mouse IgG HRP conjugate diluted in enzyme dilution buffer at 37°C for 1 h. The plates were washed again with PBST and developed by adding 100 μL of TMB substrate into each well. After 10 min, 0.3 M H_2_SO_4_ was used to stop the reaction and the optical density at 450 nm (OD_450_) was read using a PerkinElmer VICTOR X3 multilabel reader. If the test serum does not contain anti-LPS antibodies, mAb B5 would bind to LPS, resulting in color development. However, if there is anti-LPS antibodies in the test serum due to *B*. *mallei* infection, the anti-LPS antibodies will compete with mAb B5 for the epitope site and inhibit the binding of mAb B5 to LPS, resulting in an inverse proportional development of color. A conjugate control was included for the interpretation of the cELISA results. Results of cELISA were interpreted by percentage inhibition (PI) using the following formula: PI = 100 –[(OD_450_ of serum sample/OD_450_ of conjugate control) × 100].

### Complement fixation test

All sera were tested using CFT according to the OIE Manual of Diagnostic Tests and Vaccines for Terrestrial Animals [2]. Briefly, serum samples were diluted at 1:5 in CFT buffer (Institute Virion/ Serion GmbH), inactivated, diluted in two folds and mixed with the Malleus CFT antigen (ccPro, GmbH). The sera, complement and antigen were then mixed and incubated overnight at 4°C. A 2% suspension of sensitized sheep red blood cells (amboceptor from Institute Virion/ Serion GmbH) were added and the samples were incubated for 30 mins at 37°C. Samples were considered negative when 100% hemolysis occurred at a 1:5 dilution, 25–75% hemolysis were considered as inconclusive and no hemolysis was considered as positive.

### Determination of relative anti-LPS antibody titre in *B*. *mallei*-inoculated donkeys

In order to determine the relative anti-LPS antibody titre present in donkeys inoculated with live preparations of *B*. *mallei*, the serum of donkey 5 collected on day 21 post infection was selected and diluted for the generation of a relative standard curve. Briefly, the serum sample was first diluted at 1:200 in sample dilution buffer and then further diluted in 2-folds in sample dilution buffer. The diluted samples were added to microtitre plates coated with 0.01 ng of purified LPS, and incubated as described above for indirect ELISA. The presence of anti-LPS antibodies was detected with 1:10,000 anti-donkey IgG HRP conjugate (Bethyl Laboratories Inc) and developed with TMB. The OD_450_ was plotted against each dilution to generate the relative standard curve. The relative titre of anti-LPS antibodies in the original diluted sample, i.e., 1:200 serum sample, was expressed as 1.0.

Indirect ELISA was then performed for the sera samples of all the *B*. *mallei-*inoculated donkeys, including the serum sample used for generating the standard curve, as well as two seronegative donkey sera samples from Dubai. Samples were diluted 1:200 in sample dilution buffer and incubated as described above. The obtained OD_450_ readings were used to calculate the relative titre of anti-LPS antibodies in each sample using the generated standard curve.

## Results

### Generation of LPS-specific mouse monoclonal anti-LPS antibody

**Fig 1:**
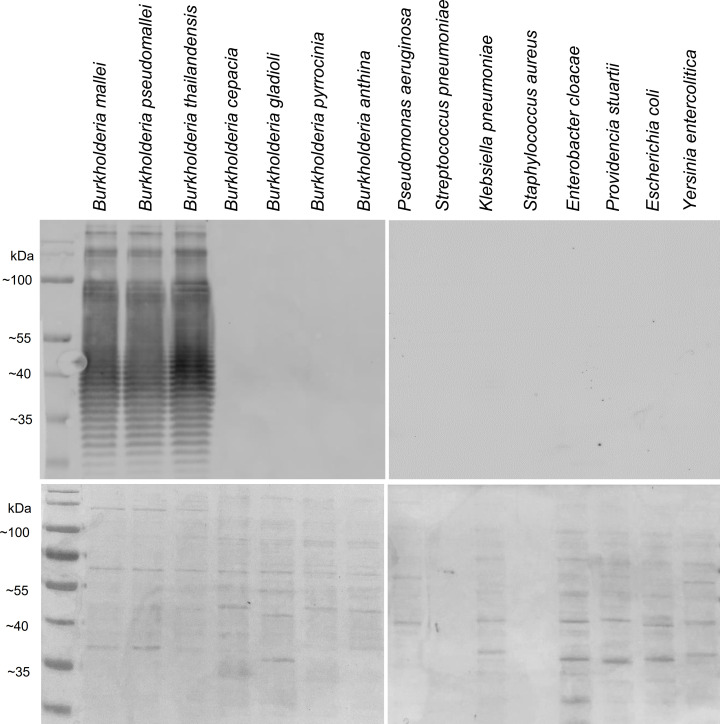
Reactivity of anti-LPS B5 to a variety of bacterial strains. Western blot (top panel) showing reactivity of the mouse monoclonal anti-LPS antibody B5 produced in this study against the bacterial lysate of seven *Burkholderia* species and eight common Gram-negative pathogens. The bottom panel shows the protein present in each lane as stained by Coomassie blue.

The mouse mAb B5 against *B*. *mallei* LPS was generated in this study using hybridoma technology. The isotype of B5 was determined to be of the subclass IgG2b with λ light chains. Western blotting showed that the mAb B5 was reactive with *B*. *mallei* LPS, with the characteristic ladder pattern of LPS displayed (Fig 1, lane 1). Reactivity was also detected for *B*. *pseudomallei* and *B*. *thailandensis*, both of which share a high degree of genetic similarity to *B*. *mallei* (Fig 1, lanes 2 and 3). No immunoreaction was detected for all the other tested *Burkholderia* species and a variety of other bacteria outside the genus *Burkholderia* (Fig 1, lanes 4 to 15; S1 Table).

### Specificity of monoclonal anti-LPS antibody B5 to LPS and optimization

**Fig 2:**
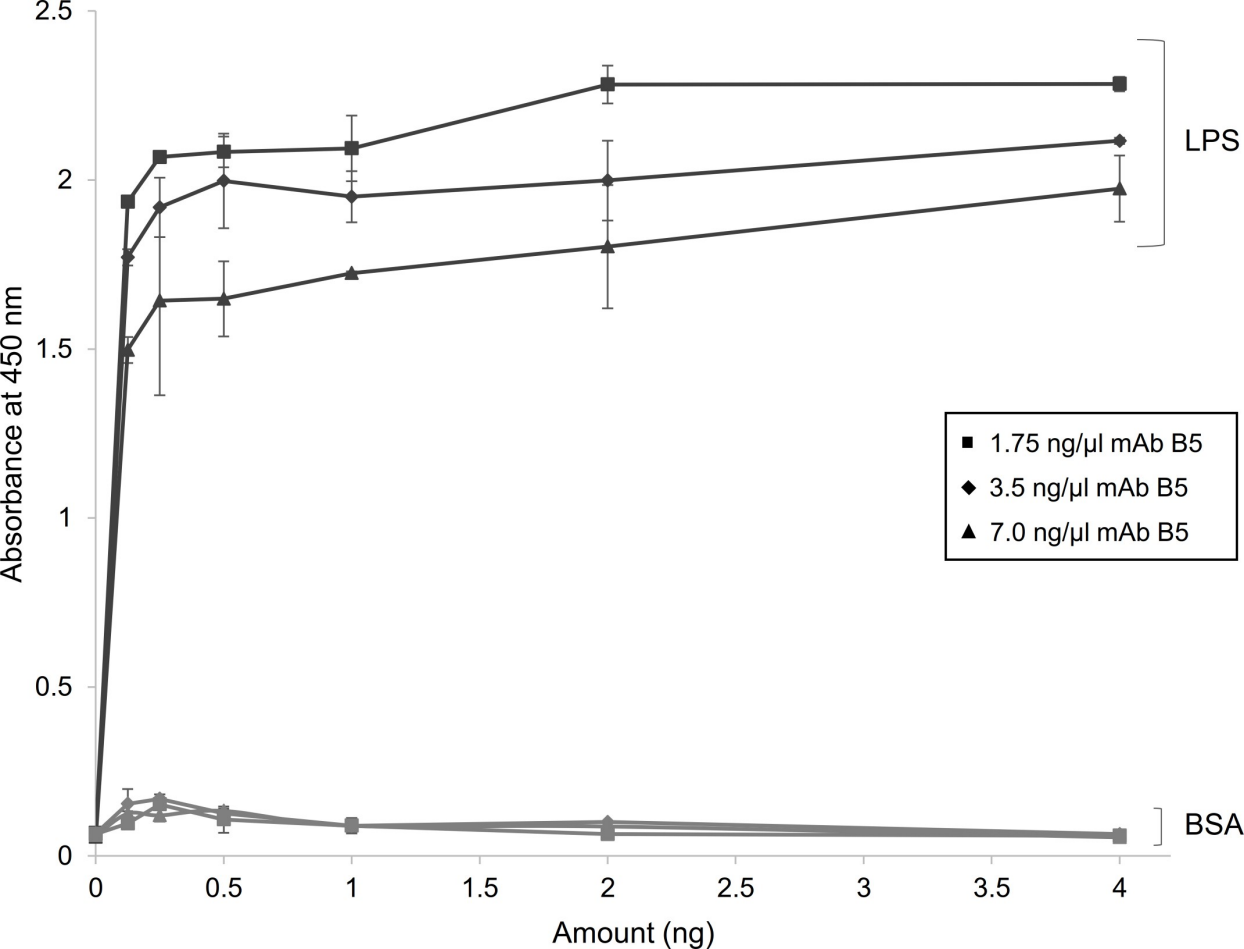
Specificity and optimization of anti-LPS B5. The specificity and optimal concentration of the mouse monoclonal anti-LPS antibody (mAb) B5 produced in this study was determined using indirect ELISA with varying amounts of *B*. *mallei* lipopolysaccharide (LPS; black lines) or bovine serum albumin (BSA; grey lines), and detecting antigen with varying concentrations (1.75, 3.5, and 7.0 ng/μL) of mAb B5.

The specificity of mAb B5 in the detection of LPS was further demonstrated using indirect ELISA. Microtiter plates were coated with serial two-fold dilutions of *B*. *mallei* LPS or BSA (0 to 4 ng) and was incubated with mAb B5 at a concentration of 1.75, 3.5 or 7 ng/μL. An increase in absorbance at 450 nm was observed with increasing LPS concentration, while no significant changes were observed with increasing BSA protein (Fig 2). This showed high specificity of mAb B5 for *B*. *mallei* LPS. The optimal concentration of LPS and antibody to use for the subsequent development of the cELISA was determined to be 2 ng of LPS and 3.5 ng/μL of mAb B5.

### Determination of the optimal serum dilution to use in the LPS-based cELISA

**Fig 3:**
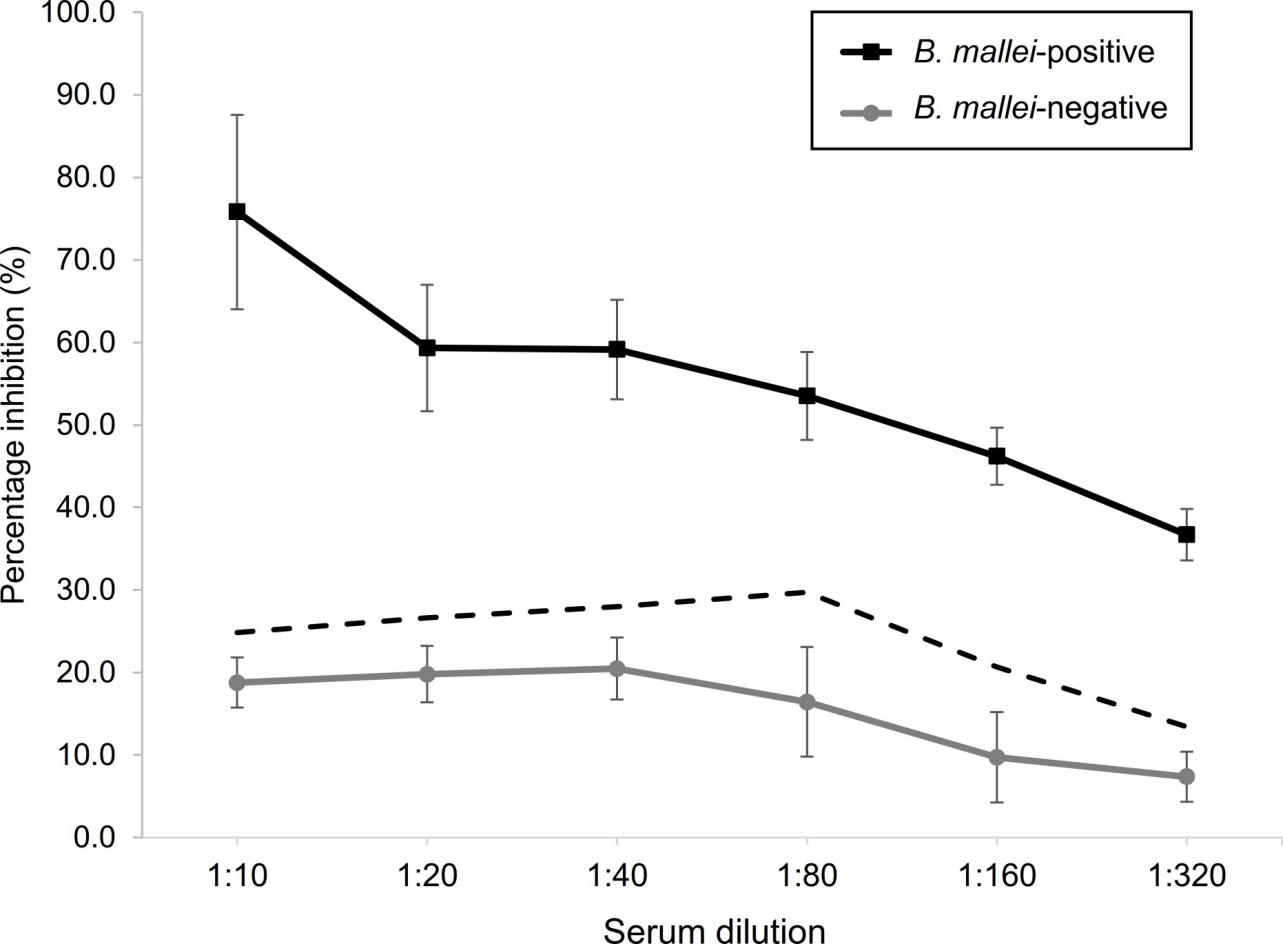
Lipopolysaccharide (LPS)-based competitive ELISA (cELISA) in the presence of 2 ng of *B*. *mallei* LPS and 3.5 ng/μL mAb B5 with varying dilutions of *B*. *mallei*-positive and negative sera. cELISA values calculated as percentage inhibition (PI) of mAb B5 binding. Serum from a glanders-seropositive horse (SE1719.4/19) from a glanders outbreak in Kuwait was used as the *B*. *mallei*-positive sample. Serum from a glanders-seronegative horse (ED18-0081-0001) from AFCD in Hong Kong was used as the *B*. *mallei*-negative sample. Dashed line indicates 2 standard deviations above the mean PI of the *B*. *mallei*-negative serum at each serum dilution.

In order to develop a cELISA for the detection of anti-LPS antibody in serum samples, the optimal dilution of serum was determined. The serum sample SE1719.4/19 from a glanders-seropositive horse in Kuwait was used as the *B*. *mallei*-positive sample, while a glanders-free seronegative horse sample ED18-0081-0001 obtained from AFCD, Hong Kong, was used as a *B*. *mallei*-negative sample. Microtiter plates coated with 2 ng *B*. *mallei* LPS was first mixed with varying dilutions of positive and negative sera from 1:10 to 1:320, followed by the addition of 3.5 ng/μL mAb B5. The binding of mAb B5 to LPS was competitively inhibited by the presence of anti-LPS antibodies in the *B*. *mallei*-positive sample (Fig 3). As the test serum became more diluted, the calculated PI also decreased, indicating that there was a lower concentration of anti-LPS antibody present which resulted in a decrease in competition with mAb B5 for the LPS epitope. No significant decrease in the calculated PI was observed for BSA-coated wells in the presence of a more diluted test serum. The PI values of the *B*. *mallei*-positive sample at each dilution remained more than 2 SDs above the mean of the seronegative serum as presented by the dashed line in Fig 3, indicating the ability of the cELISA to distinctly separate and determine a sample as *B*. *mallei-*positive or negative. The largest difference between the PI values of the positive and negative samples were observed at the serum dilution of 1:10. However, taking into account that in actual situations, the volume of test serum available may be limited, hence, the next best serum dilution of 1:20 was selected for use in the subsequent development and evaluation of the cELISA.

### Determination of the cutoff value of the LPS-based competitive ELISA

The optimal conditions for the LPS-based cELISA was determined to be the coating of microtiter plates with 2 ng *B*. *mallei-*LPS, using of sample serum at 1:20 dilution, and the addition of 3.5 ng/μL mAb B5. These conditions were subsequently used for the cELISA.

**Fig 4:**
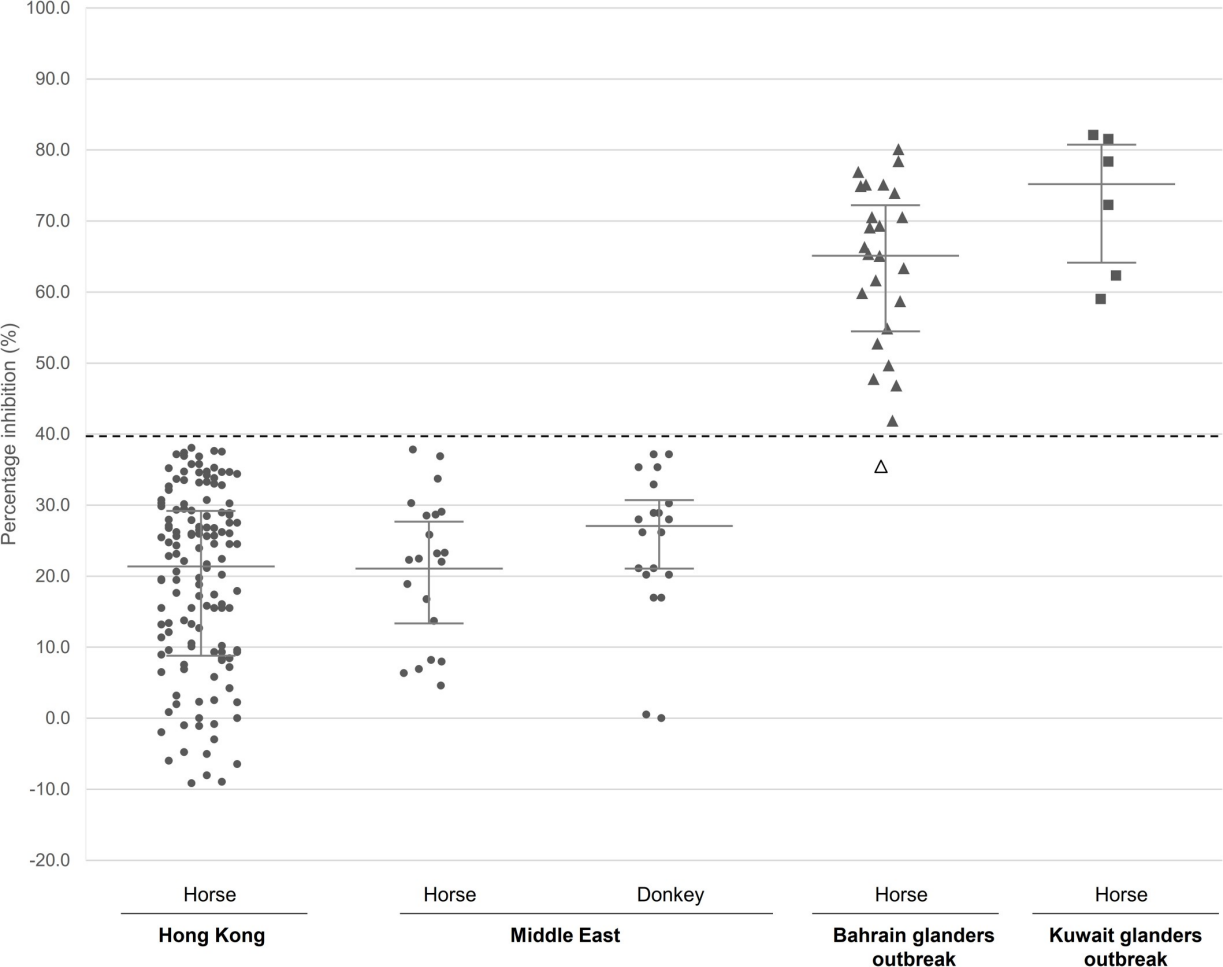
Evaluation of lipopolysaccharide-based competitive ELISA (cELISA) using horse and donkey sera obtained from Hong Kong and the Middle East (Dubai, Bahrain and Kuwait). Data indicate the distribution of cELISA values calculated as percentage inhibition (PI) of mAb B5 binding. The defined cELISA cutoff value (PI of 39.6%) is indicated by the dashed line. Solid lines represent the median and interquartile range. Circle, seronegative sera from glanders-free horses and donkeys; triangle, horse seropositive sera from a glanders outbreak in Bahrain; square, horse seropositive sera from a glanders outbreak in Kuwait; empty triangle, horse seropositive serum from a glanders outbreak in Bahrain which was tested as negative by the developed LPS-based cELISA.

The cutoff value of the cELISA was determined using serum samples from 136 glanders-free seronegative horses in Hong Kong (Fig 4). The calculated PI values ranged from -9.2 to 38.6% with a mean PI of 20.7%. At 2 SD above the mean PI, the calculated cutoff value was defined as 45.9%, meaning that sera with PI values above 45.9% was considered positive for anti-LPS antibody. However, from our results, we observed that all calculated PI values from the seronegative samples were in fact placed below 39.6% inhibition, which corresponds to 1.5 SD above their mean PI. This indicated that, using a representative number of negative controls, this lower inhibition value could also be considered a threshold of the cELISA. We therefore determined the cutoff value of the LPS-based cELISA as 39.6%, with calculated PI values between 39.6 to 45.9% defined as ‘weakly positive’. Serum samples with a PI value of less than 39.6% were considered negative for the presence of anti-LPS antibodies. For animals with serum samples that had calculated PI values within the ‘weakly positive’ range, the collection and retesting of another serum sample from the animal was recommended to further confirm the positive presence of anti-LPS antibodies.

### Detection of glanders infection in animals challenged with *B*. *mallei*

**Fig 5:**
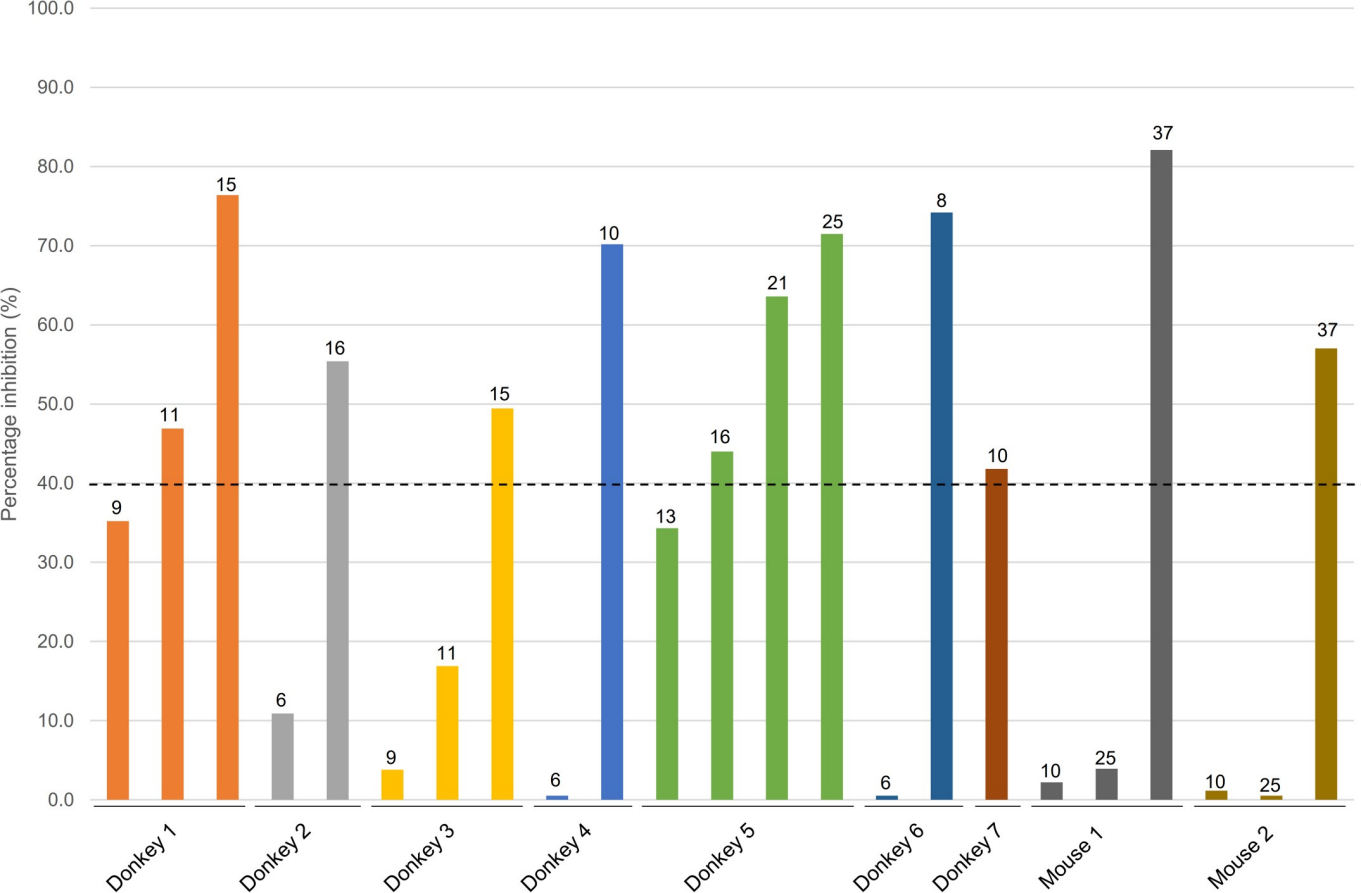
Using the lipopolysaccharide-based competitive ELISA (cELISA) to detect anti-LPS antibodies in seven donkeys and two mice inoculated with *B*. *mallei*. The donkeys were challenged via the following routes: oral injection at inoculum of 1.0 × 10^9^ CFU/mL (donkeys 1 and 2), oral injection at inoculum of 1.0 × 10^8^ CFU/mL (donkey 3), direct feeding through water (donkey 4), direct feeding through feed (donkey 5), intranasal spray (donkey 6), and subcutaneous injection (donkey 7). The two mice (mouse 1 and 2) were inoculated twice with non-viable *B*. *mallei* via intraperitoneal injection. Numbers on top of bars indicate the day post infection (donkey) or the day after the first inoculation (mouse) on which sera was collected. Data indicate cELISA values calculated as the percentage inhibition (PI) of mAb B5 binding. The defined cELISA cutoff value (PI of 39.6%) is indicated by the dashed line.

The diagnostic sensitivity of the developed LPS-based cELISA was assessed using sera from seven donkeys and two mice inoculated with live and non-viable preparations of *B*. *mallei*, respectively (Fig 5). An increasing trend in PI values above the defined cELISA cutoff of 39.6% was first observed in donkey sera collected from as early as 8 dpi (infected by intranasal route) up to 16 dpi (infected by direct feeding through feed), suggesting the positive detection of anti-LPS antibodies generated by successful *B*. *mallei* infection. This increasing trend in antibody level was sustained until at least 25 dpi. A similar increase in PI values above the defined cELISA cutoff was also observed in mice sera collected at 37 days after the first inoculation, suggesting the positive detection of anti-LPS antibodies. This observation was in line with vaccination studies of mice where a good IgG response was observed in vaccinated mice two weeks after the second inoculation [16].

### Comparison of serodiagnostic detection of anti-LPS antibody by competitive ELISA with relative anti-LPS antibody titre in *B*. *mallei-*inoculated donkeys

The positive and negative detection of anti-LPS antibodies in the sera of donkeys challenged with *B*. *mallei* were compared with the calculated relative titres of anti-LPS antibodies present (Table 1). The sera of two seronegative donkeys from Dubai were included to represent control titre levels in the absence of glanders infection. The CFT results for each sample were also included for comparison. We observed that the cELISA assay was able to detect the presence of anti-LPS antibody when the relative titre of antibody is ≥0.295, whereas the CFT can detect a relative titre of ≥0.145. Of the 17 samples, neither tests had false positive results for any of the samples, however, the cELISA was falsely negative for 3 samples, while CFT was falsely negative for 1 sample. We confirmed that the cELISA was able to detect the presence of anti-LPS antibody in the sera sample and its performance was relatively comparable to that of the CFT.

**Table 1:** Comparison of test results of the LPS-based competitive ELISA developed in this study (cELISA) and the complement fixation test (CFT) for the serodiagnosis of glanders in donkeys inoculated with *B*. *mallei* with the relative titre of anti-LPS antibody present.

Donkey	Route of infection (inoculum dosage)	Days post infection	Relative titre of anti-LPS antibody*	Test Result	
				cELISA	CFT^†^
1	Oral injection (1.0 × 10^9^ CFU/mL)	9	0.415	-	1:20+
		11	0.321	+	1:40+
		15	0.382	+	1:80+
2	Oral injection (1.0 × 10^9^ CFU/mL)	6	0.037	-	-
		16	0.295	+	1:80+
3	Oral injection (1.0 × 10^8^ CFU/mL)	9	0.145	-	1:20+
		11	0.305	-	1:40+
		15	0.669	+	1:40+
4	Direct feeding through water (2.0 × 10^8^ CFU/mL)	6	-0.029	-	-
		10	0.482	+	1:40++
5	Direct feeding through feed (4.0 × 10^8^ CFU/mL)	13	0.599	-	1:320+
		16	0.692	+	1:320++
		21	0.820	+	1:160++
		25	0.528	+	1:80++
6	Intranasal spray (1.0 × 10^8^ CFU/mL)	6	0.038	-	-
		8	0.394	+	-
7	Subcutaneous injection (1.0 × 10^2^ CFU/mL)	10	0.447	+	1:10++
Negative	N/A	N/A	-0.143	-	-
Negative	N/A	N/A	-0.180	-	-

*calculated by a standard curve generated using dilutions of the serum collected on day 21 post infection of donkey 5

^**†**^-, 100% hemolysis; +, 25% hemolysis; ++, 50% hemolysis; +++, 75% hemolysis; ++++, 0% hemolysis

### Evaluation of LPS-based competitive ELISA using sera from glanders outbreak samples

The sensitivity and specificity of the LPS-based cELISA was further evaluated using 21 seronegative horse sera and 20 seronegative donkey sera from Dubai as well as 31 serologically positive horse sera from glanders outbreaks in Bahrain and Kuwait (Fig 4). The PI values of the seronegative horse and donkey sera ranged from 4.6% to 37.8% and 0 to 37.3% inhibition, respectively. On the other hand, the PI values of the seropositive sera from the Bahrain and Kuwait glanders outbreak ranged from 35.4 to 80.1% and 59.0 to 82.1% inhibition, respectively. One seropositive serum from the Bahrain outbreak (PI = 35.4%) was tested as a false-negative by the assay.

Overall, based on the defined cELISA cutoff value of 39.6% inhibition, 97.2% (35/36) of the seropositive sera were tested as positive, with 2/36 having a PI value within the ‘weakly positive’ range, while 100% (41/41) of the seronegative sera were tested as negative. These results agreed with our expectation that samples with calculated PI values above 39.6% were positive for the presence of anti-LPS antibodies. Our evaluation established the developed LPS-based cELISA as highly sensitive (97.2%) and specific (100%) for the detection of anti-LPS antibody in sera samples.

## Discussion

Serodiagnosis for glanders requires the detection of *B*. *mallei* antibodies in serum samples of infected animals. In general, ELISA that detects the presence of antibodies in a special kind of animal necessitates the availability of secondary antibodies against that group of animals. However, secondary antibodies against some groups of animals may not be readily available. For example, when we detected influenza virus in a giant panda in a local amusement park two years ago [17] and would like to examine the corresponding immune response in the giant panda, we found that secondary antibodies against giant pandas are not available in the market. Therefore, we had to resort to use the haemagglutinin inhibition test but could not perform more specific serological tests. However, such non-specific serological test is only available for a limited number of infections. For those infections without non-specific serological tests, secondary antibodies against that specific animal would need to be generated. For example, when we tried to develop an ELISA for serodiagnosis of aspergillosis in falcons, we generated anti-falcon secondary antibodies by immunizing guinea pigs using purified falcon IgY [18], as anti-falcon antibodies were not available. Moreover, even if these secondary antibodies are available, setting up such tests for *n* types of animals would mean purchasing *n* types of secondary antibodies, which has major cost implications. Since *B*. *mallei* is known to infect different kinds of animals [1,3,19–20], it would be optimal to develop a one-kit-for-multiple-animal serological test which can serve to detect *B*. *mallei* antibodies in a variety of animals.

In this study, we developed a highly sensitive LPS-based cELISA for serodiagnosis of *B*. *mallei* infections. LPS is an important outer membrane component of Gram-negative bacteria that contributes to the structural integrity of the bacteria [21]. It also has a crucial role in bacterial-host interaction by eliciting strong host innate immune responses [22], which leads to the production of anti-LPS antibodies. As a primary component that is encountered by the host immune system, it serves as an early indicator of acute infection. Thus, LPS are important antigens that are often used in serological diagnostic tests for a variety of bacteria. Studies have shown that *B*. *mallei* and *B*. *pseudomallei* LPS are also potent activators of human Toll-like receptor 4 and stimulates human macrophage-like cells [23–24]. Interestingly, melioidosis patients who survive the infection are found to have higher levels of anti-LPS antibodies than those who succumb to the disease [25]. For the present study, we extracted LPS from a clinical strain of *B*. *mallei* of an infected horse and used it to immunize mice for the generation of mAb B5 against the LPS. The cELISA developed in this study allows the detection of these anti-LPS antibodies in animal sera through competition with LPS-mAb B5 interactions, which in turn provides serodiagnosis of *B*. *mallei* infection. Although the mAb B5 antibody was raised against *B*. *mallei* LPS, we observed that it was also reactive towards the LPS of *B*. *pseudomallei* and *B*. *thailandensis* (Fig 1). *B*. *mallei* and *B*. *pseudomallei* are phylogenetically very similar and have nearly identical 16S ribosomal DNA sequences [26], while *B*. *thailandensis* is recognized as an avirulent close relative of *B*. *pseudomallei* which can be distinguished by its capacity to assimilate arabinose, keto-gluconate and adonitol and inability to utilize erythritol and dulcitol as sole carbon sources [27]. Studies have shown that *B*. *mallei* and *B*. *pseudomallei* produce structurally similar LPS anchored to their outer membranes [28], and are antigenically closely related to each other [29] and to *B*. *thailandensis* [30]. Similar LPS ladder patterns are also observed in SDS-PAGE analyses of *B*. *pseudomallei*, *B*. *mallei*, and *B*. *thailandensis* LPS but which differed from those of other *Burkholderia* species [30]. The cross-reactivity of mAb B5 to *B*. *pseudomallei* and *B*. *thailandensis* is a limitation of the cELISA to distinguish between the LPS of these species. However, in terms of its use to diagnose disease, glanders is primarily a disease of equines, whereas melioidosis is more commonly observed in domesticated animals such as goats, sheep and pigs [31], which are resistant to *B*. *mallei*, and humans, which is susceptible to *B*. *mallei* but is not a common infection. Thus it should be possible to define the disease based on the type of animal that is infected. As for *B*. *thailandensis*, it is generally considered avirulent with limited reports of infection by this bacterium in humans [32–34], hence would not have caused serious disease as observed for glanders or melioidosis.

The optimal amount of LPS (2 ng per well) and concentration of mAb B5 (3.5 ng/μL) to use in this assay was determined by using indirect ELISA to compare the absorbance values at varying amounts and concentrations. This represented the optimal conditions where stable interactions between LPS and mAb B5 were observed. Application of these conditions to develop the cELISA resulted in the selection of the serum dilution of 1:20 for further evaluation of the assay. This next best dilution ensured that animals can still be tested even when the volume of serum that can be collected is limited. The cutoff value of 39.6% was determined using sera obtained from horses that were imported for horse racing in Hong Kong. By protocol, these animals undergo strict quarantining at special stabling facilities after arrival and are required to be tested by our local government authority to ensure that they are free of equine diseases, thus were useful glanders-free serum samples for determining the cutoff. After fixing the cutoff value, the specificity of the cELISA was further evaluated using 41 CFT confirmed seronegative samples from Dubai. Results showed that the PI of all these samples were 0 to 37.8%, indicating a 100% specificity.

The cELISA is also highly sensitive in detecting *B*. *mallei* antibodies. The sensitivity of the LPS-based cELISA was first assessed using sera from donkeys challenged with *B*. *mallei*. High PI values were measured from the donkeys inoculated by the oral and nasal route. This coincides with the natural transmission route of *B*. *mallei* between animals, which is facilitated by close contact, inhalation and ingestion of contaminated materials [1–2]. On the other hand, a lower PI value was detected from the donkey which was infected by the non-natural transmission route of subcutaneous inoculation. In fact, the PI value fell within the range of ‘weakly positives’, indicating the detection of only a low titer of anti-LPS antibody in this serum sample. This agrees with our expectation that when the calculated PI is within the range of 39.6 to 45.9%, the animal is likely to be positive for *B*. *mallei* infection, though the immune response of the animal to the bacteria may have been low or the animal may have only been recently exposed to the bacteria, resulting in low antibody levels. However, challenge studies with a larger number of animals will need to be performed to further verify linkage between the infection route and resulting antibody levels. The sensitivity of the cELISA was also assessed with sera collected from mice that were inoculated simulating a vaccination. Previous studies have shown that mice inoculated with non-viable cell preparations of *B*. *mallei* resulted in immune responses producing an increase in IgA, IgG and IgM levels [16]. Correspondingly, the cELISA was able to detect an increase in anti-LPS antibody in the inoculated mice in the same timeframe as observed in the vaccination study. In addition, by measuring the relative anti-LPS titre present in the *B*. *mallei*-challenged donkey sera, we confirmed that the cELISA accurately detects the presence of anti-LPS antibody in the sera samples and its performance is relatively comparable to that of the CFT, which is the current ‘gold standard’ for the diagnosis of glanders infection. Although the sensitivity and specificity of both tests are similar, the cELISA is less time-consuming and does not involve laborious preparations with expensive reagents. The cELISA also requires less serum volume, which may be limiting in some instances. The development of improved methodologies and faster approaches for diseases of high concern is particularly beneficial for areas where the pathogen is endemic.

The cELISA was further evaluated with horse sera from glanders outbreaks in Bahrain [9–10] and Kuwait [11]. The outbreak in Bahrain was a result from horses imported from Syria via Kuwait. Clonality of the outbreak was confirmed by high-resolution genotyping and comparative whole genome analysis [9] and all the positive sera were confirmed by CFT. Our results showed that 97.2% of the 36 CFT positive sera were tested positive by the cELISA, confirming a high sensitivity of the assay. Such high sensitivity and specificity of the cELISA makes it a user-friendly and inexpensive assay for laboratory diagnosis of glanders for different animals in veterinary laboratories.

## Supporting information

S1 Table Bacterial strains used in this study to test the reactivity of anti-LPS antibody B5 against the bacterial lysate of seven *Burkholderia* species and eight common Gram-negative pathogens. (DOCX)
