# Supplementary material for: Development of a sensitive competitive enzyme-linked immunosorbent assay for serodiagnosis of Burkholderia mallei, a Tier 1 select agent
Source: PLoS Negl Trop Dis. 2021 Dec 21;15(12):e0010007. doi: 10.1371/journal.pntd.0010007 (PMC8691619; doi:10.1371/journal.pntd.0010007)
Supplement: S1 Table — (DOCX) [file pntd.0010007.s001.docx]

**S1 Table. Bacterial strains used in this study to test the reactivity of anti-LPS antibody B5 against the bacterial lysate of seven *Burkholderia* species and eight common Gram-negative pathogens**

| **Bacterial species** | **Strain** | **Source** | **Origin/Reference** |
| --- | --- | --- | --- |
| *Burkholderia mallei* | MB1731 | Equine choana | [1] |
| *Burkholderia pseudomallei* | BC334 | Patient isolate | This study |
| *Burkholderia thailandensis* | Bt1 | Patient isolate | [2] |
| *Burkholderia cepacia* | LMG1222 | *Allium cepa* | [3] |
| *Burkholderia gladioli* | LMG2216 | *Gladiolus* sp. | [4] |
| *Burkholderia pyrrocinia* | LMG14191 | Soil | [2] |
| *Burkholderia anthina* | LMG20980 | Soil | [5] |
| *Pseudomonas aeruginosa* | ATCC27853 | Patient isolate | [6] |
| *Streptococcus pneumoniae* | PW927 | Patient isolate | This study |
| *Klebsiella pneumoniae* | PW3290 | Patient isolate | This study |
| *Staphylococcus aureus* | ATCC25923 | Patient isolate | [7] |
| *Enterobacter cloacae* | 2337-2 | Patient isolate | This study |
| *Providencia stuartii* | 6187-1 | Patient isolate | This study |
| *Escherichia coli* | ATCC25922 | Patient isolate | [8] |
| *Yersinia enterolitica* | PW5588 | Patient isolate | This study |

**References:**

1. Altemann D, Bauerfeind R, Wernery U. Pathogenesis of glanders in experimentally infected feral donkeys using different infection routes and doses. J Equine Vet Sci. 2012;32:S82.
2. Lau SK, Lam CW, Curreem SO, Lee KC, Chow WN, Lau CC, et al. Metabolomic profiling of *Burkholderia pseudomallei* using UHPLC-ESI-Q-TOF-MS reveals specific biomarkers including 4-methyl-5-thiazoleethanol and unique thiamine degradation pathway. Cell Biosci. 2015;5:26.
3. Palleroni NJ, Holmes B. *Pseudomonas cepacia* sp. nov., nom. rev.. Int. J. Syst. Bacteriol. 1981;31:479-481.
4. Yabuuchi E, Kosako Y, Oyaizu H, Yano I, Hotta H, Hashimoto Y, et al. Proposal of *Burkholderia* gen. nov. and transfer of seven species of the genus *Pseudomonas* homology group II to the new genus, with the type species *Burkholderia cepacia* (Palleroni and Holmes 1981) comb. nov. Microbiol Immunol. 1992;36(12):1251-75.
5. Vandamme P, Henry D, Coenye T, Nzula S, Vancanneyt M, LiPuma JJ, et al. *Burkholderia anthina* sp. nov. and *Burkholderia pyrrocinia*, two additional *Burkholderia cepacia* complex bacteria, may confound results of new molecular diagnostic tools. FEMS Immunol Med Microbiol. 2002;33(2):143-9.
6. Medeiros AA, O'Brien TF, Wacker WE, Yulug NF. Effect of salt concentration on the apparent in-vitro susceptibility of *Pseudomonas* and other gram-negative bacilli to gentamicin. J Infect Dis. 1971;124 Suppl:S59-64.
7. Fretheim K, Granum PE, Vold E. Influence of generation temperature on the chemical composition, antioxidative, and antimicrobial effects of wood smoke. J. Food Sci. 1980;45:999-1002.
8. Minogue TD, Daligault HA, Davenport KW, Bishop-Lilly KA, Broomall SM, Bruce DC, et al. Complete Genome Assembly of *Escherichia coli* ATCC 25922, a Serotype O6 Reference Strain. Genome Announc. 2014;2(5):e00969-14.
